# Synthesis and crossover reaction of TEMPO containing block copolymer via ROMP

**DOI:** 10.3762/bjoc.6.59

**Published:** 2010-06-01

**Authors:** Olubummo Adekunle, Susanne Tanner, Wolfgang H Binder

**Affiliations:** 1 Institute of Chemistry, Faculty of Natural Sciences II (Chemistry and Physics), Martin-Luther University, Halle-Wittenberg, von Danckelmannplatz 4, D-06120 Halle, Germany

**Keywords:** block copolymer (BCP), crossover reaction, MALDI, NEOLYST^™^, ROMP

## Abstract

We report on the block copolymerization of two structurally different norbornene monomers (±)-*endo*,*exo*-bicyclo[2.2.1]-hept-5-ene-2,3-dicarboxylic acid dimethylester (**7**), and (±)-*endo*,*exo*-bicyclo[2.2.1]-hept-5-ene-2,3-dicarboxylic acid bis(1-oxyl-2,2,6,6-tetramethyl-piperidin-4-yl) ester (**9**) using ruthenium based Grubbs’ type initiators [(PCy_3_)_2_Cl_2_Ru(benzylidene)] **G1** (PCy_3_ = tricyclohexylphosphine), [(H_2_IMes)(PCy_3_)Cl_2_Ru(benzylidene)] **G2** (H_2_IMes = 1,3-bis(mesityl)-2-imidazolidinylidene), [(H_2_IMes)(py)_2_Cl_2_Ru(benzylidene)] **G3** (py = pyridine or 3-bromopyridine) and Umicore type initiators [(PCy_3_)_2_Cl_2_Ru(3-phenylinden-1-ylidene)] **U1** (PCy_3 _= tricyclohexylphosphine), [(H_2_IMes)(PCy_3_)Cl_2_Ru(3-phenylinden-1-ylidene)] **U2** (H_2_IMes = 1,3-bis(mesityl)-2-imidazolidinylidene), [(H_2_IMes)(py)Cl_2_Ru(3-phenylinden-1-ylidene)] **U3** (py = pyridine or 3-bromopyridine) via ring opening polymerization (ROMP). The crossover reaction and the polymerization kinetics were investigated using matrix assisted laser desorption ionization mass spectroscopy (MALDI-TOF) and nuclear magnetic resonance (NMR), respectively. MALDI showed that there was a complete crossover reaction after the addition of 25 equivalents of the second monomer. NMR investigation showed that **U3** gave a faster rate of polymerization in comparison to **U1**. The synthesis of block copolymers with molecular weights up to *M*_n_ = 31 000 g/mol with low polydispersities (*M*_w_/*M*_n_ = 1.2) is reported.

## Introduction

Block copolymers are macromolecules composed of linear or non-linear arrangements of chemically different polymeric chains. If the different blocks are incompatible, a rich variety of well defined self-assembled structures both in bulk and selective solvents arises [1]. The synthetic approach to block copolymers has been widely discussed [1] and achieved extensively via living polymerization methods. Thus, besides acyclic diene metathesis polymerization (ADMET) [2], ring opening metathesis polymerization (ROMP) [3–5] is another type of olefin metathesis polymerization that can be used for the synthesis of block copolymers.

**Scheme 1:**
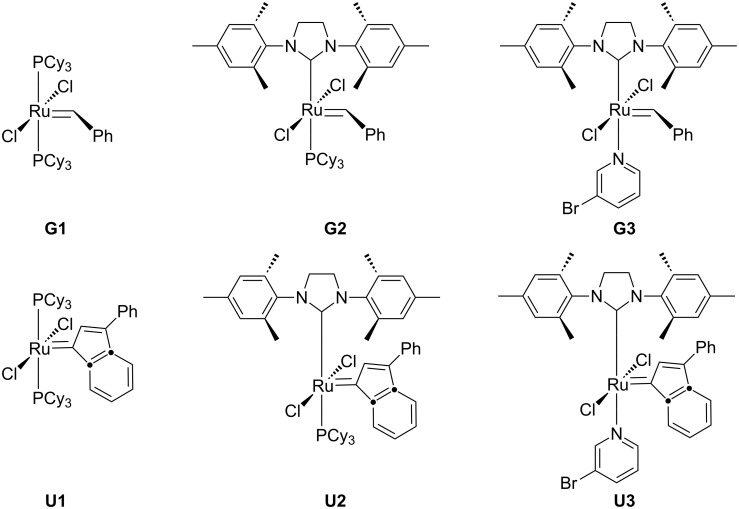
Grubbs **G1**–**G3** and Umicore **U1**–**U3** catalyst.

Early examples of catalysts for ROMP were based on molybdenum alkylidene catalysts, however, the true breakthrough of the method was hampered by the restricted functional group tolerance of Schrock initiators due to their sensitivity towards protic solvents and air [6]. With the advent of the Grubbs’ catalyst **G1** (see Scheme 1) and related complexes as initiators, polymerization reactions can now not only be performed in protic media but also without rigorous exclusion of molecular oxygen. However, these advantages are hampered by the considerable lower activity of catalysts such as **G1** when compared with Schrock’s molybdenum catalysts [7–9]. Often, the polydispersity indices of the resulting polymers obtained with initiator **G1** are large with values ranging between 1.3 and 1.5 arising from an unfavorable rate of initiation (*k*_i_) relative to propagation (*k*_p_) as well as considerable secondary metathesis (backbiting). Grubbs’ second generation catalyst **G2** displays an activity comparable to the Schrock type initiators. It exhibits a higher functional group tolerance than **G1**, but initiation by catalyst **G2** is often slow as a result of the slow dissociation of the phosphine group, sometimes limiting its application in polymer synthesis. Alternatively, Grubbs’ third generation catalyst **G3** introduced by Grubbs et al. [10] has an ultrafast initiating ruthenium benzylidene. The rate of reaction of **G3** with ethyl vinyl ether thus is six orders of magnitude higher than for **G2** [10], leading to a faster initiation and often lower polydispersities of the resulting polymers.

**Scheme 2:**
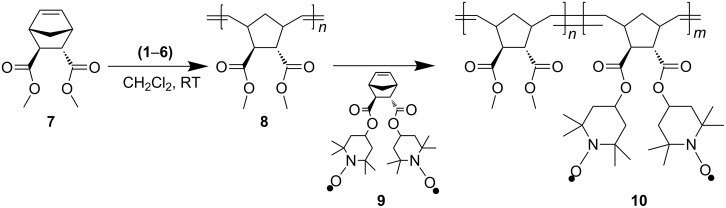
Synthetic pathway to BCP-A*_n_*T*_m_* using Grubbs’ (**1**–**3**) and Umicore (**4**–**6**) type catalysts.

Recently, structural variations of **G1**–**G3** catalysts generated a new series of catalysts **U1**–**U3** bearing indenyl-carbenes instead of benzylidene-carbenes. These new catalysts are now commercially available and are well known as the Umicore catalysts (NEOLYST^™^). However, their synthetic profile with respect to the synthesis of block copolymers is largely unexplored. As we recently have reported extensively on the use of ROMP methods in blockcopolymer synthesis [11–13], either via direct copolymerization or coupled to postmodification methods via azide/alkyne-“click”-chemistry [14–17], the crossover reaction of more complex monomers remains the crucial factor in achieving defined BCP’s with low polydispersities. In a recent example, the crossover reaction of various monomers with the Grubbs’ type catalysts **G1**–**G3** was studied in detail via MALDI mass spectrometry [11], revealing a more detailed picture of the crossover reaction (Scheme 2). Thus mass spectrometry could often demonstrate insufficient crossover reactions between monomers of different reactivity such as monomer A **7** and monomer T **9**, despite a low polydispersity when the crossover reaction was monitored by conventional GPC methods [11]. A semi-quantification method of the respective spectra allowed a good correlation between the rate-constants of initiation and propagation of the different monomers.

The current publication describes the synthesis of block copolymers A*_n_*T*_m_* composed of monomers **7** and **9**, initiated via the catalysts **U1**–**U3**, as well as mass spectrometric investigations of the crossover reactions via MALDI methods. The incorporation of the free radical **9** into block copolymer is an important contribution in the generation of polymers for reversible charge storage materials, as monomer **9** can accept or donate electrons reversibly.

## Results and Discussion

The polymerization of monomer **7** was investigated using catalysts **U1**–**U3** (see Table 1). Basically, the catalyst **U3** showed good polymerization behavior, furnishing the homopolymers (entries 2, 3) with excellent control of chain length and low polydispersities (*M*_w_/*M*_n_ = 1.2). The catalysts **U1** and **U2** gave poor results (see entries 4 and 5) presumably due to slow initiation and fast polymerization, which is in accord with the structurally similar catalysts **G1** and **G2** (see Scheme 1). Similarly, the polymerization of monomer T **9** was investigated, which gave good results with the catalysts **G2**, **G3**, and **U3** (entries 6, 7, and 8). The other catalysts **G1**, **U1**, and **U2** did not yield good polymerization results (data not shown) and were therefore not considered further for the synthesis of the respective block copolymers.

**Table 1:** Overview of polymerization result of monomer A **7** and monomer T **9** with the catalysts **U1**–**U3** and **G1**–**G3**.

entry	polymer	1st monomer	2nd monomer	catalyst	molecular weight (g/mol)		PDI
					GPC	calculated	

1	Homo-A_15_^a^	Mon-A	—	**G3**	3700	3100	1.1
2	Homo-A_15_^a^	Mon-A	—	**U3**	2700	3150	1.2
3	Homo-A_25_^a^	Mon-A	—	**U3**	4410	5250	1.2
4	Homo-A_50_^b^	Mon-A	—	**U1**	8500	10500	1.2
5	Homo-A_50_^b^	Mon-A	—	**U2**	418000	10500	1.4
6	Homo-T_20_	Mon-T	—	**G2**	12900	9800	1.7
7	Homo-T_100_^b^	Mon-T	—	**G3**	48800	49000	1.3
8	Homo-T_50_^b^	Mon-T	—	**U3**	24100	24500	1.3
9	BCP-A_15_T_1_^a^	Mon-A	Mon-T	**U3**	3200	3640	1.1
10	BCP-A_15_T_2_^a^	Mon-A	Mon-T	**U3**	4020	4130	1.2
11	BCP-A_15_T_4_^a^	Mon-A	Mon-T	**U3**	4400	5110	1.1
12	BCP-A_25_T_1_^a^	Mon-A	Mon-T	**U3**	5100	5740	1.1
13	BCP-A_25_T_2_^a^	Mon-A	Mon-T	**U3**	5500	6230	1.1
14	BCP-A_25_T_4_^a^	Mon-A	Mon-T	**U3**	6100	7770	1.1
15	BCP-A_15_T_1_^a^	Mon-A	Mon-T	**G3**	4600	3640	1.1
16	BCP-A_15_T_2_^a^	Mon-A	Mon-T	**G3**	4900	4140	1.1
17	BCP-A_15_T_5_^a^	Mon-A	Mon-T	**U3**	5680	7700	1.1
18	BCP-A_25_T_25_	Mon-A	Mon-T	**U3**	17000	17500	1.1
19	BCP-A_50_T_50_	Mon-A	Mon-T	**U3**	31300	35000	1.2
20	BCP-A_10_T_10_	Mon-A	Mon-T	**U3**	7300	7000	1.1
21	BCP-A_20_T_20_	Mon-A	Mon-T	**U3**	13400	14000	1.1

^a^The polymer was synthesized for MALDI analysis. ^b^The experiment was performed for kinetic measurements by taking samples every 5 minutes.

**Figure 1:**
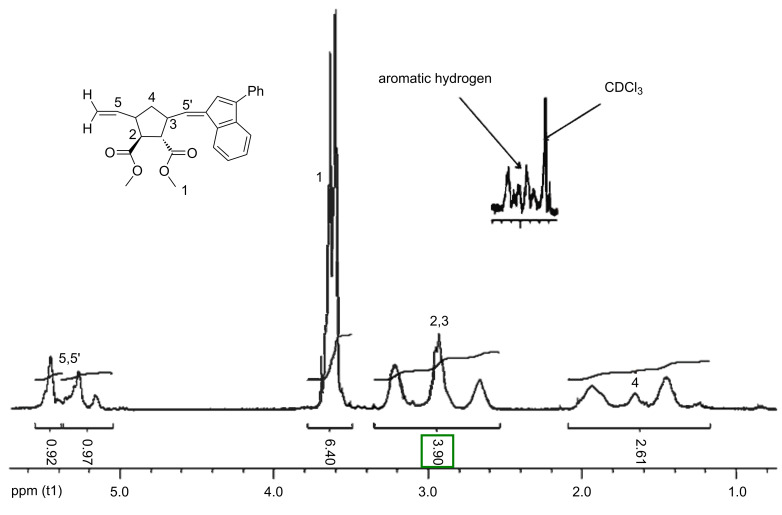
^1^H NMR spectrum of the homopolymer A_50_ synthesized with the catalyst **U3**.

**Figure 2:**
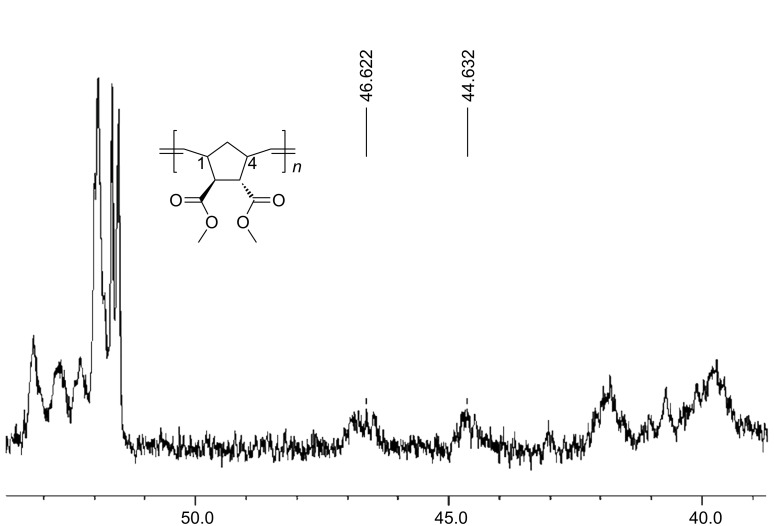
^13^C NMR spectrum of the homopolymer A_50_ synthesized with catalyst **U3**.

**Figure 3:**
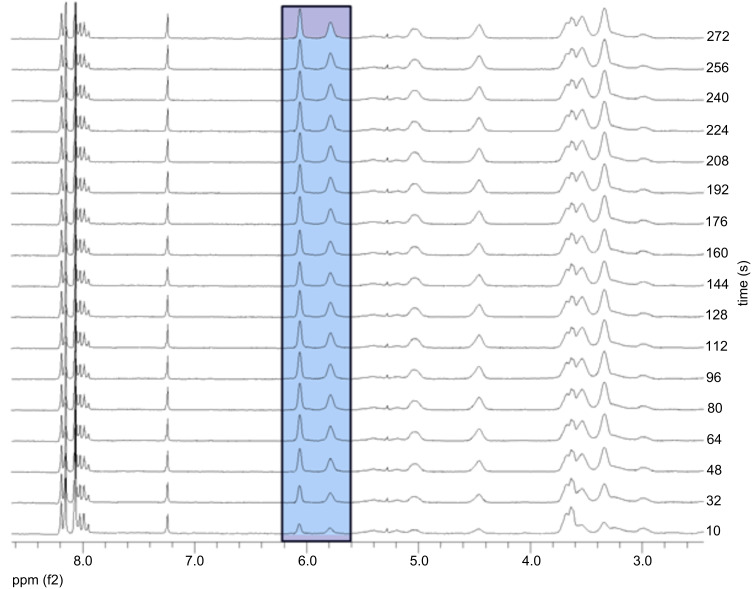
Kinetic progress monitored via ^1^H NMR spectroscopy of the polymerization of monomer A **7** with catalyst **U1**; [**7**]/[**U1**] = 20/1.

**Figure 4:**
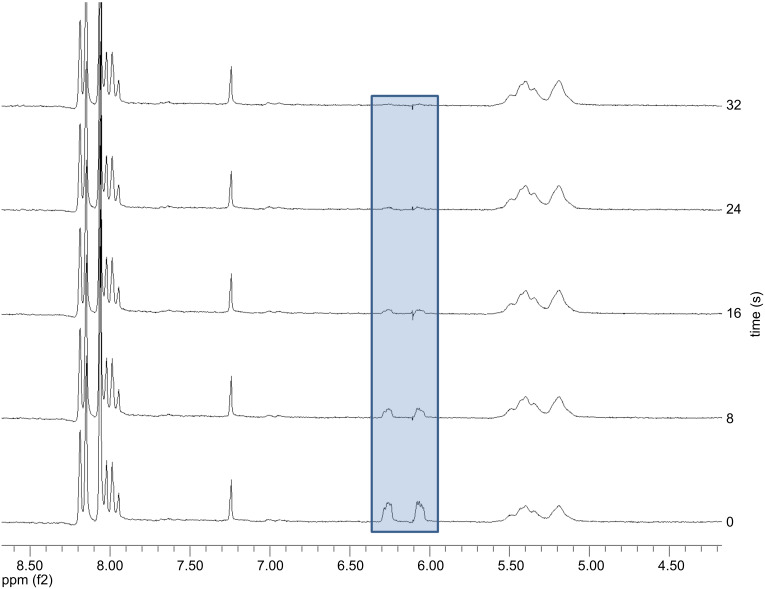
Kinetic progress monitored via ^1^H NMR spectroscopy of the polymerization of **7** with catalyst **U3**; [**7**]/[**U3**] = 20/1.

**Figure 5:**
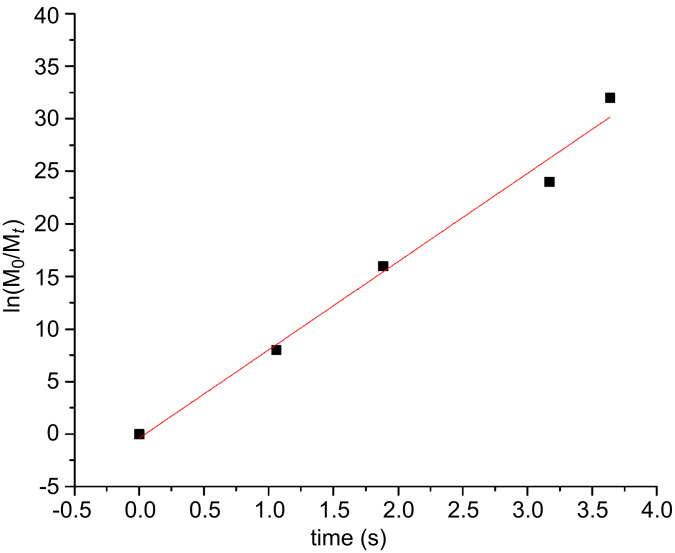
ln([M]_0_/[M]*_t_*) vs. time (*t*) plot obtained from ^1^H NMR spectra for homopolymer-A_50_ using catalyst **U3**; [**7**]/[**U3**] = 20/1.

The ^1^H NMR spectrum (Figure 1) of the respective homopolymer (A_50_) clearly shows the expected resonances together with the resonances of the indenyl-moieties of the initiator-structure. The spectrum further revealed that the unsaturated polymer exhibited no stereoregularity (*cis*/*trans* ~ 50/50 see the peaks at 5.2 and 5.4 ppm in Figure 1) which is in accordance with results reported in the literature [18]. Figure 2 shows the relevant region of the ^13^C NMR spectrum, with the approximately equal peak intensities indicating an equal *m*:*r* ratio. Thus the polymerization yields the respective polymer, although in poor yields which is underlined when monitoring the kinetics of the polymerization of monomer A **7** using catalysts **U1** and **U3** (see Figure 3, Figure 4 and Figure 5). As expected in accordance with the known polymerization reactions of the respective Grubbs’ type catalysts, catalyst **U3** polymerizes significantly faster (the polymerization reaction is complete after ~20 seconds) whereas the polymerization initiated with catalyst **U1** takes significantly longer and never yields significant amounts of the homopolymer (yield < 10 %).

As the polymerization kinetics of **7** using catalyst **U3** could not be monitored effectively with GPC because it was too fast (50 units were polymerized in less than 1 minute), the kinetics were monitored by NMR. NMR measurements were conducted every 8 seconds and the result showed that the polymerization was complete within ~20 seconds as shown in Figure 4 and Figure 5. The rate of polymerization (*k*_p_*)* was calculated by integrating the peaks (6.25 and 6.07 ppm) corresponding to the alkene protons of monomer A **7** as they disappeared. Plotting ln([M]_0_/[M]*_t_*) vs. time (*t*) gave a straight line as shown in Figure 5 and indicated linear chain growth. The slope of the straight line was divided by the initial initiator concentration [I]_0_ assuming a first order kinetics which gave *k*_p_ as 18.5 l·mol^−1^·s^−1^.

**Figure 6:**
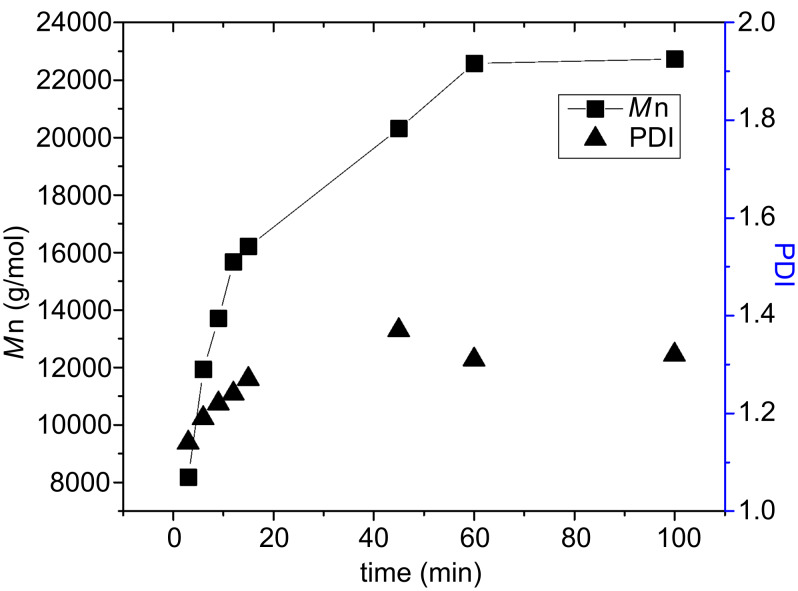
*M*_n_ vs. time (*t*) kinetic plot and *M*_w_/*M*_n_ (PDI) of the polymerization of monomer T **9** with catalyst **U3**; [**9**]/[**U3**] = 50/1.

As monomer T **9** is a free stable radical, the progress of its polymerization with catalyst **U3** could not be monitored by ^1^H NMR spectroscopy. Therefore, the conventional method of following the *M*_n_ vs. time (*t*) profile was carried out as shown in Figure 6. Chain growth with a maximum polydispersity of *M*_w_/*M*_n_ ~1.3 was observed, clearly proving the high precision of this type of polymerization reaction.

**Figure 7:**
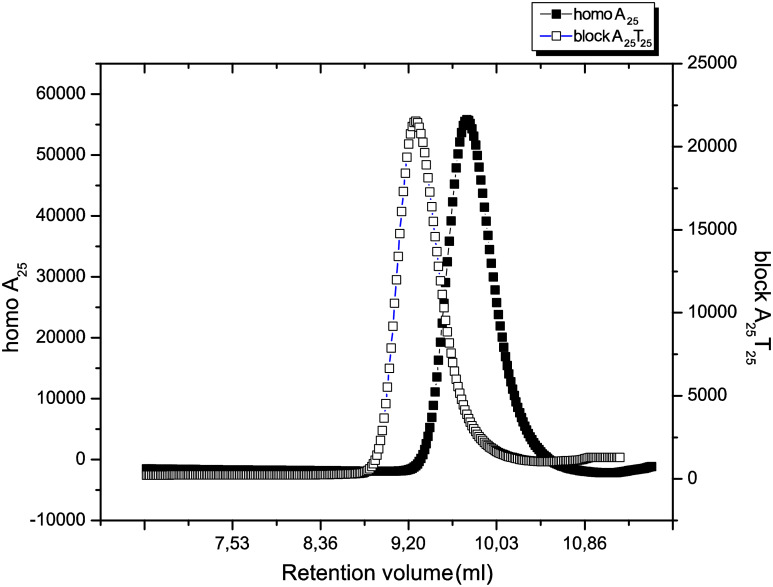
GPC trace of the block copolymer A_25_T_25_ synthesized with catalyst **U3**.

As the polymerization of both, monomer A **7** and monomer T **9** proceeded well with catalyst **U3**, the synthesis of the BCP was achieved by use of this initiating system to yield the respective BCP-A_10_T_10_, A_20_T_20_, A_25_T_25_ and A_50_T_50_ with the expected molecular weight and with low polydispersity (see Table 1, entries 18–21). The GPC traces of A_25_ block and the A_25_T_25_ block copolymer are shown in Figure 7, indicating the expected shift in the retention time after addition of monomer T **9** after all of the monomer A **7** had been consumed.

**Figure 8:**
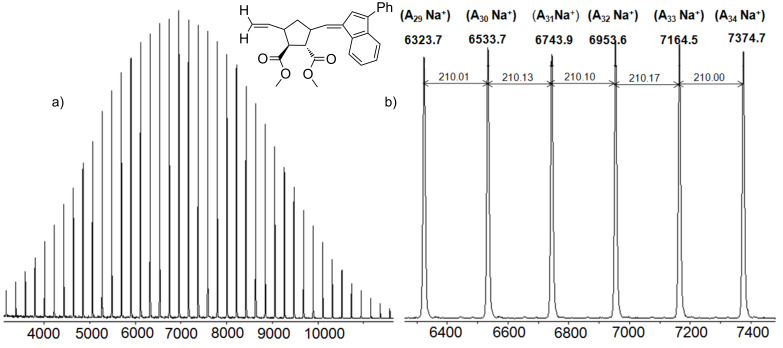
MALDI-TOF mass spectra of homopolymer A_25_ **4** synthesized with catalyst **U3**: (a) full spectrum, (b) expansion.

In order to achieve a deeper insight into the exact nature of the crossover reaction when changing from monomer A **7** to monomer T **9** with catalyst **U3**, the respective reaction was monitored according to our previous methods using MALDI mass spectrometry [11]. Thus homopolymer A_25_ was initiated with catalyst **U3** and subsequently reacted with 1, 2, 5 and 25 equivalents of monomer T after all of the monomer A **7** had been consumed. The respective samples were then quenched with ethyl vinyl ether, and subsequently analyzed by MALDI-TOF mass spectrometry and GPC. The GPC results are shown in Table 1, entries 9–14 and 18–21, indicating that with increasing amount of added monomer T an equal increase of *M*_n_ can be observed. However, in order to check for the detailed composition of the reaction mixture, MALDI spectra were measured. As shown in Figure 8, homopolymer A_25_ can be desorbed well in MALDI, featuring the respective A*_n_*Na^+^-ions as a pure series. Thus the homopolymer A*_n_* can serve as molecular probe for the subsequent desorption of the individual A*_n_*T_1, 2, 5_-species.

**Figure 9:**
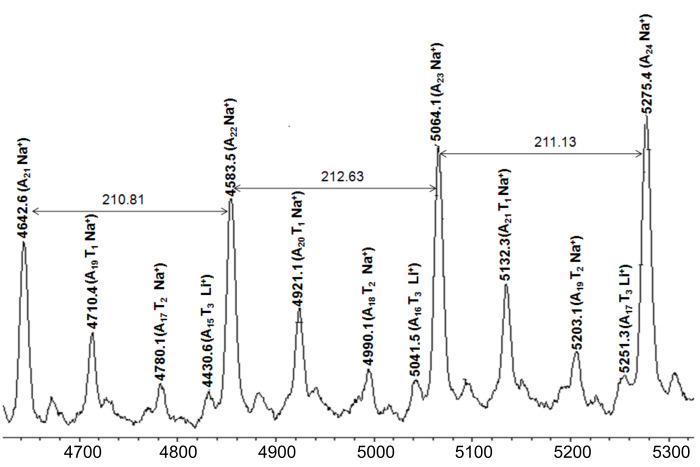
MALDI-TOF mass spectrum of BCP-A_25_T_1_ synthesized with catalyst **U3**.

The MALDI spectrum of the crossover reaction of A_25_ with exactly one equivalent of monomer T **9** using **U3** as initiator is shown in Figure 9. Thus, together with the still present A*_n_*-series (visible as A*_n_*Na^+^-series), the respective crossover species A*_n_*T_1_, and A*_n_*T_2_ can be seen as the respective Na^+^-ions. These results demonstrate that a large amount of A*_n_*-species did not participate in the crossover reaction, since due to the fast polymerization of monomer T **9**, it was rapidly consumed, leading to A*_n_*T_2_-species and its respective higher homologues.

**Figure 10:**
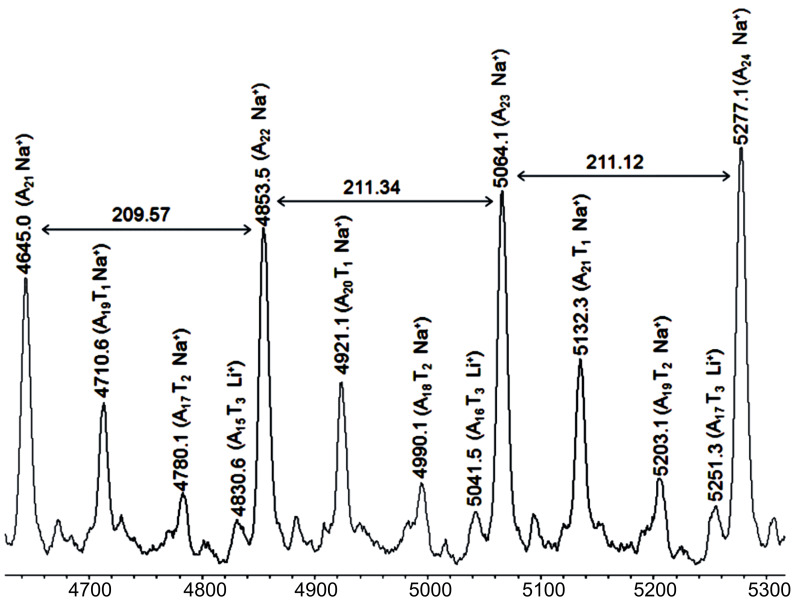
MALDI-TOF mass spectrum of BCP-A_25_T_2_ **13** synthesized with catalyst **U3**.

**Figure 11:**
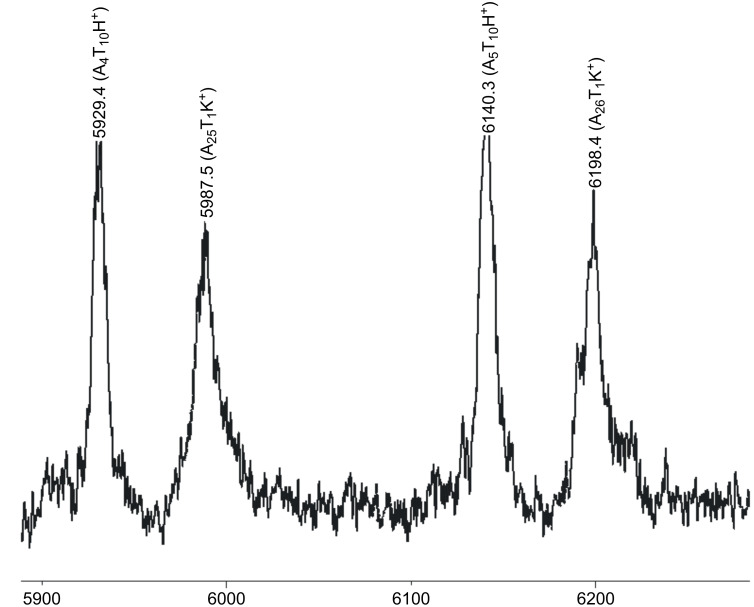
MALDI-TOF mass spectrum of BCP-A_25_T_25_ synthesized with catalyst **U3**.

The respective MALDI spectrum of the crossover reaction of the homopolymer A_25_ with exactly two equivalents of monomer T **9** is shown in Figure 10. Again, a significant amount of homopolymer A*_n_* (visible as A*_n_*Na^+^-series) is present in the reaction mixture, the respective crossover-species A*_n_*T_1_, and A*_n_*T_2_ can be seen as the respective Na^+^-ions. Additionally, the respective series A*_n_*T_3_ is visible, indicative of the further chain growth process after the crossover reaction. Again, despite the excess of T*_n_*-species a large amount of A*_n_*-species did not participate in the crossover reaction due to the fast polymerization of monomer T **9**. MALDI spectra of a further series of block copolymers A_25_T_5_ and A_25_T_25_ was carried out in order to check for the presence of residual homopolymer A_25_ in the polymer mixture (see Figure 11). We could not detect any residual homopolymer in either of these final samples. As it is known from our previous investigations, that the homopolymer A*_n_* in MALDI is desorbed preferentially by a factor of 13 with respect to the A_25_T*_n_*-species, this now indicates a complete cross-over reaction and thus the successful preparation of block copolymers A*_n_*T*_m_* via this methodology. Basically, this synthetic approach now allows the synthesis of AT type BCP’s with high precision and chain length control up to molecular weights of ~31000 g/mol.

## Conclusion

The synthesis of new block copolymers containing free radical centers within one block via ROMP has been described. MALDI analyses especially provide a detailed picture of the crossover reaction. Basically, the NEOLYST^™^ catalysts are comparable to the well known Grubbs’ catalysts, indicating a similar profile of initiation and propagation. However, the catalyst **U3** is especially a highly potent catalyst for ROMP and displays a broad profile of tolerance against functional groups within the monomer, enabling the successful synthesis of block copolymers containing free-radical species in high densities.

## Experimental

### General Remarks

**Solvents/Reagents/Materials:** Catalysts **G1**, **G2** and **G3** were obtained from Sigma-Aldrich. Catalysts **U1**, **U2** and **U3** were obtained as gifts from the Umicore chemical company. All reagents used for the synthesis of norbornene monomers **7** and **9** were obtained from Sigma-Aldrich Chemical Co. (Germany) and used as received without further purification unless otherwise indicated. Bicyclopentadiene (100%), fumaric acid (99+%), thionyl chloride (99+%, Fluka), pyridine (99.8%), methanol and 4-hydroxy-2,2,6,6-tetramethyl-piperidin-1-oxyl (TEMPOL) were obtained from Sigma-Aldrich and used without further purification. Dichloromethane (CH_2_Cl_2_) was freshly distilled over CaH_2_ and degassed with argon prior to use. Other solvents such as hexane and ethyl acetate were used after distillation.

**Instrumentation: **^1^H NMR spectra were recorded on a Varian Gemini 400 MHz FT-NMR spectrometer, and MestRec (4.9.9.9) was used for data interpretation. The polymerization kinetics of the polymerization reactions with both catalysts **U1** and **U3** were measured at 25 °C on a 200 MHz FT-NMR spectrometer using CDCl_3_ as a solvent. GPC analysis was performed on a Viscotek VE2001 system with THF as the eluant at a flow rate of 1 ml/min and an injection volume of 100 µL. Polystyrene standards were used for conventional external calibration using a Viscotek VE3580 refractive index detector. Positive ion MALDI-TOF (matrix-assisted laser desorption ionization time-of-flight) measurements were performed on a Bruker Autoflex-III instrument equipped with a smart ion beam laser. Measurements were carried out in linear and reflector mode. Samples were prepared from THF solution by mixing matrix (20 mg/ml), polymer (20 mg/ml), and salt (20 mg/ml solution) in a ratio of 100:10:1. Dithranol (1,8-dihydroxy-9(10*H*)-anthracetone, Aldrich 97%) was used as the matrix. Sodium trifluoroacetate (Aldrich, 98%), silver trifluoroacetate (Aldrich, 99.99%) or lithium trifluoroacetate (Aldrich, 99.8%) were added for ion formation, with sodium trifluoroacetate as the optimal salt for obtaining the highest S/N ratio.

### Monomer synthesis

5-Norbornene-*endo*,*exo*-2,3-dicarboxylic acid dimethylester, monomer A **7** was synthesised according to reference [11], 5-norbornene-*endo, exo*-2,3-dicarboxylic acid bis(1-oxyl-2,2,6,6-tetramethyl-piperidin-4-yl) ester, monomer T **9**, was prepared according to references [19–20].

### Synthesis of homopolymers A_15_ and T_20_

Monomer A **7** (50.0 mg, 0.23 mmol) dissolved in 1 ml of CH_2_Cl_2_ was introduced into a heated and argon flushed glass tube equipped with a magnetic stirring bar. A solution of catalyst **U3** (11.8 mg, 0.015 mmol) dissolved in 1 ml of CH_2_Cl_2_ was then added. After 5 min of stirring at room temperature, the total consumption of monomer A **7** was confirmed by thin layer chromatography (TLC). The reaction was then quenched with 5 drops of cold ethyl vinyl ether, and the resulting polymer purified by column chromatography (SiO_2_). The homo-polymerization of monomer T **9** was carried out in the same manner with catalyst **G2**. Homopolymers (A*_n_*) with different chain lengths (*n* = 15, 50, 25) with the catalysts **G3**, **U1**, **U2** and **U3** as initiators were also synthesized using the same procedure by adopting the required polymerization times.

### Block copolymer syntheses

The synthesis of block copolymers (A*_n_*-*b*-T*_n_*) was carried out analogously to methods developed previously in our laboratory [14,16]. For example the synthesis of BCP-A_50_T_50_ was performed by sequential addition of monomers. Monomer A **7** (15 mg, 0.071 mmol) dissolved in 1 ml of CH_2_Cl_2_ was introduced into a heated and argon flushed glass tube equipped with a magnetic stirring bar. To this solution, catalyst **G3** (1.26 mg, 0.0014 mmol) dissolved in 1 ml of CH_2_Cl_2_ was then added. The mixture was allowed to stir at room temperature for 1 h until all of the monomer A **7** was consumed as confirmed by GPC and TLC. Subsequently, monomer T **9** (35 mg, 0.071 mmol) dissolved in 1 ml of CH_2_Cl_2_ was then added to the above reaction mixture and stirred for 2 h at room temperature until all of monomer T **9** was consumed, as confirmed by GPC and TLC. The polymerization was quenched by the addition of cold ethyl vinyl ether. The polymer was isolated by column chromatography (SiO_2_) (eluent: DCM).

### Kinetic experiments

A pyrene stock solution was prepared from 70 mg of pyrene dissolved in 2 ml of CDCl_3_. Monomer A **7** (20.83 mg, 0.099 mmol) dissolved in CDCl_3_ (0.2 ml) was first introduced into the NMR tube and then the pyrene stock solution (0.2 ml) was added. Before adding the initiator solution, the ratio of the monomer to the internal standard was determined by NMR. On the basis of this value, the monomer concentration at *t* = 0 was determined. A solution of the catalyst **U3** (1.48 mg, 0.0019 mmol), dissolved in CDCl_3_ (0.2 ml) (in case of catalyst **U1** (1.83 mg, 0.0019 mmol)) dissolved in CDCl_3_ (0.2 ml) was added via a syringe to yield the desired monomer to initiator ratio. After shaking, the tube was inserted into the NMR spectrometer, and the decrease in the monomer with respect to time was monitored by integrating the resonance peaks at 6.27 and 6.07 ppm. For determination of the monomer concentration at *t* = 0 and the monomer consumption, the corresponding signals at 6.27 and 6.07 ppm from monomer A **7** compared with the one at 8.20 ppm from the internal standard pyrene were integrated. The time between the addition of the initiator solution and the first measurement was added to the first measuring point.
